# Use of lactulose as a prebiotic in laying hens: its effect on growth, egg production, egg quality, blood biochemistry, digestive enzymes, gene expression and intestinal morphology

**DOI:** 10.1186/s12917-023-03741-x

**Published:** 2023-10-16

**Authors:** Hassan S. Elkomy, Ivan I. Koshich, Sahar F. Mahmoud, Magda I. Abo-Samaha

**Affiliations:** 1 Poultry Breeding and Production, Animal Husbandry and Animal Wealth Development, Faculty of Veterinary Medicine, Alexandria University, Edfina, Beheira, 22758 Egypt; 2 Moscow State Academy of Veterinary Medicine and Biotechnology Named After K.I. Skryabin, 23, Academician Skryabin Street, Moscow, 109472 Russia; 3 Histology and Cytology Department, Faculty of Veterinary Medicine, Damanhur University, Damanhur, 22511 Egypt

**Keywords:** Vetelact, Lactulose, Layer, OCX-36, OVAL, OC-116, OCX-32

## Abstract

**Background:**

The rising popularity of eggs as an alternative source of protein to meat has led to significant increase in egg consumption over the past decade. To meet the increasing demand for eggs, poultry farmers have used antibiotics to treat infections and, to some extent, promote growth and egg production in raising layer. However, the emergence and global spread of antibiotic resistant bacteria has now necessitated antibiotic-free poultry farming. As alternatives to antibiotics, prebiotics are feed additives that can be used to improve the growth and laying performance of poultry which positively impacts their performance and general health. In this study we evaluated the effect of lactulose, formulated as Vetelact, on body weight, egg production, egg quality, blood biochemical parameters and expression of genes associated with reproductive performance in laying hens.

**Results:**

Vetelact supplementation improved egg weight, egg production as well as egg quality. Following Vetalact supplementation, the levels of total bilirubin, total protein, globulin and phosphorus were increased, while the activities of alkaline phosphatase and lipase enzymes were increased compared to control. Vetelact at 0.10 ml/kg body weight upregulated OCX-36, OVAL, CALB1, OC-116, OCX-32 and IL8 transcripts while downregulating the transcription of Gal-10, PENK and AvBD9. At this optimal inclusion rate of Vetalect, histomorphologic analyses of intestinal tissue showed increased villi length with more goblet cell distribution and obvious mucus covering a surface, increase in the depth of intestinal crypts produce digestive enzymes, as well as more developed muscle layer that promote improved nutrient absorption.

**Conclusion:**

Vetelact at a dose of 0.10 ml/ kg body weight was effective in improving productive performance of laying hens. Adding lactulose (0.10 ml/ kg body weight) to layer diet is recommended to promote growth and improve egg laying performance in antibiotics-free poultry production.

## Background

The Chicken layer industry is an economically important animal production system. The rising popularity of eggs as an alternative source of protein to meat has led to significant increase in egg consumption over the past decade. To meet the increasing demand for eggs, poultry farmers have used antibiotics to treat infections and, to some extent, promote growth and egg production in raising layer [1]. These include cyclic peptides (e.g., bacitracin), ionopohores (e.g., monensin, narasin), streptogramins (e.g., virginiamycin), orthosomycins (e.g., avilamycin), and macrolides (e.g., tylosin, spiramcycin) [1, 2]. However, the prolonged use of antibiotic growth promoters in poultry farms increases the population of antibiotic-resistant bacteria [1]. Therefore, research into alternate options to antibiotics has increased to maintain or enhance the performance of farm animals [3]. The natural alternatives to antibiotics include probiotics, prebiotics, symbiotics, organic acids, essential oils, enzymes and plant extracts [4]. Prebiotics have been reported as one of the feed additives that could improve the growth and laying performance of poultry, with. positive impacts on their performance and general health [5, 6]. Prebiotics are indigestible feed additives that promote the growth and activity of beneficial microorganisms like *Bifidobacteria* and *Lactobacillus* in the intestine [7]. The presence of these organisms has been associated with enhanced performance and nutrient digestibility in the host [8]. Lactulose (4-*O*-β-d-galactopyranosyl-d-fructose) is a non- digestible carbohydrate that is currently considered as a prebiotic [9, 10]. It is a synthetic disaccharide [11] that is used clinically as a laxative [12]. At low doses, lactulose serves as prebiotic [12, 13], inhibits the activity of proteolytic bacteria and promotes the growth of *Lactobacillus* and *Bifidobacterium* [14, 15]. Lactulose ingestion has been shown to have benefits in humans, mice, rats, sows, and pigs by increasing probiotic and putrefactive bacteria, decreasing the potential pathogens, and subsequently reducing the activity of pro-carcinogenic enzymes such as azoreductase, and 7-alpha-dehydroxylase [16–18]. Dietary lactulose (0.1 or 0.2%) improves the productivity Of broilers and reduce the level of *Escherichia coli* in their excreta [19]. Broiler body weight gain (BWG) and feed conversion ratio (FCR) improved (from d 0 to 21) when supplemented with increasing amounts of lactulose. Additionally, intestinal morphology improved with selective stimulation intestinal microflora and increased cecal short chain fatty acids (SCFAs) concentrations [20].

Prebiotics have been supplemented in the diet of young and traditional layer hens [21, 22]. Adding of oligofructose-type prebiotics and inulin in the layer's diet boosted egg production and increased the egg weight as compared to the control group [23]. Oligofructose and inulin have a positive effect on layer hens due to their direct correlation with the rate of mineral absorption [24]. Supplementing these compounds in diets markedly improved eggshell strength, eggshell weight, total ash, serum calcium levels, tibia phosphorus, and calcium levels. In broiler chicken, BWG was increased by increasing the amount of lactulose in the diet from zero to 0.5% [25]. Additionally, lactulose-containing feed additives have a positive effect on the biochemical composition of blood, metabolic processes and the natural resistance in broiler [26].

There are limited reports about using lactulose in broiler chickens [20, 25, 26] have been mentioned, however, the effect of lactulose on laying performance, egg quality and genes related to reproduction and immunity have not been reported. In the present study, we evaluated the effects of lactulose in the form of Vetelact on body weight, egg production, egg quality, blood biochemical parameters and expression of genes associated with reproductive performance in laying hens.

## Materials and methods

### Animal care

The experimental procedures were carried out according to Directive 2010/63/EU of the European Parliament and of the Council of 22 September 2010 on the protection of animals used for scientific purposes. The study was conducted at Moscow State Academy of Veterinary Medicine and Biotechnology–MVA by K. I. Skryabin, Moscow, Russian Federation, while the histopathology was performed in Faculty of Veterinary Medicine, Damanhur university, Egypt.

### Experimental design

One week prior to the experiment, Hisex brown layers with similar body weight and egg-laying performance were selected. A total of 120 Hisex brown layer chickens (35 weeks old) were purchased from GENOFOND OOO, 141315, Sergiev Posad, st. Maslieva, Moscow, Russia. Birds were distributed randomly into 4 groups (30 chickens/treatment) with three replicates per treatment (10 chickens per replicate). All groups were fed standard diet (basic ration) that was prepared according to NRC recommendations [27]. The nutrient level of the basal diet is presented in Table 1. According to the manufacturer, the Vetelact product (NEC Agrovetzashchita SP, Russia) contains at least 50% lactulose and other carbohydrates. Vetelact was added in drinking water [28, 29] at 0.00, 0.05, 0.10 and 0.15 ml/kg live body weight, in Groups 1 (control), 2, 3 and 4 respectively. Before supplementing Vetelact, birds were weighed and the required amount was determined for each treatment and then added to water. We followed manufacturers’ instructions for Vetelact supplementation, which was done for 4 weeks starting at 35 weeks and continued till the end of the experiment at 39 weeks. Feed and water were allowed ad libitum for birds.

**Table 1:** Nutrient levels of the basal diet

Nutritional parameters	Levels
Corn	40.00
Wheat	21.37
Soybean meal	15.20
Sun flower meal	5.00
Soybean, full fat	6.00
Oil	1.69
Limestone	8.28
Dicalcium phosphate	1.60
Common salt	0.35
Vitamin and mineral premix^a^	0.15
DL-Methionine	0.25
DL-Treonine	0.06
Antioxidant	0.05
Total	100.00
Calculated results	
Energy Kcal/kg	2620
Crude Protein + Phytase %	16.60
crude fat	2.50
crude fiber %	5.12
Calcium %	3.40

^a^Vitamin and mineral premix provided per kilogram of diet: vitamin A, 9000 IU; vitamin D3, 3000 IU; vitamin E, 15 mg; vitamin K3, 10 mg; vitamin B1 4 mg; vitamin B2, 8 mg; vitamin B6, 5mg; vitamin B12, 0.025mg; niacin, 50 mg; pantothenic acid, 20 mg; folic acid, 20 mg; biotin, 0.25 mg; choline, 175 mg, i canthaxanthin 250 mg, manganese, 100 mg; zinc, 150 mg; iron, 100 mg,; cupper, 20 mg; iodine, 1.5 mg; cobalt, 0.5 mg; selenium, 0.2 mg; molybdenum, 1mg; magnesium, 50 mg

Laying hens were housed in battery cages with single hen per cage (commercial compact type wire cages, 50 × 44 × 46 cm) equipped with nipple drinkers and trough feeders. Laying hens were maintained in the experimental room with windows and received additional artificial light to provide 16 h of light and 8 h of dark.

### Productive performance parameters

Eggs weights were individually determined weekly by electronic scales (ME-R 326AFU, Mercury Equipment, China). Laid eggs were counted daily, and the egg laying rate was calculated weekly. We collected a total of 360 eggs (90/ treatment) at end of experiment when the hens were 39 weeks old to determine egg quality. Shape index (SI) was calculated according to [30] SI = [width/Length] × 100 by using digital caliper. For sampling, each egg was weighed and broken, and the height of the thick albumen and egg yolk were measured within a tripod micrometer. The albumen and yolk were separated, and only yolk was weighed. Additionally, yolk height and diameter were measured with the aid of a calipertor allow the calculation of the yolk index, Yolk index = (height of yolk)/ (average diameter of yolk)*100 [31]. Haugh units (HU) were calculated from the formula [HU = 100 log (H − 1.7EW^0.37^ + 7.57)] [32]. Eggshell thickness (mean of 3 different sides of eggs, μm) was measured with same micrometer.

### Blood collection

At the end of the experiment (39 weeks), blood samples (9 samples per treatment) were collected by brachial vein puncture into plane vacutainer tubes and centrifuged at 2500 × *g* for 10 min. Serum was aspirated into a 2.5 mL centrifuge tube and stored at − 20 ^O^C until analysis for metabolites, proteins, minerals and enzymes.

### Serum biochemical indices

Laboratory testing of blood samples was carried out at the International Laboratory of Molecular Genetics and Genomics of Poultry, Moscow State Academy of Veterinary Medicine and Biotechnology–MVA by K. I. Skryabin. The serum biochemical indices, including calcium (Ca), phosphorus (P), blood urea nitrogen (BUN), creatinine (CREA), glucose (GLU), Cholesterol (CHOL), total bilirubin (TBIL), total protein (TP), albumin (ALB), globulin (GLOB), albumin/ globulin (ALB/GLOB) and enzymes parameters including alkaline phosphatase (ALKP), alanine aminotransaminase (ALT), Gamma-glutamyltransferase (GGT), amylase, lipase were assayed using IDEXX Catalyst One Chemistry Analyzer Chem 17 CLIP assay kits (Maine, U.S).

### Gene expression analysis

Birds were euthanized by cervical dislocation. Samples of uterus and cecum were collected. To assess the expression of genes associated with production and immunity in chickens, total RNA was isolated from tissue fragments. Total RNA was manually isolated from the samples using the RNeasy Midi Kit (QIAGEN, Germany) according to the manufacturer’s instructions. The quality control of total RNA was carried out on a Qubit 3.0 fluorimeter using the QubitTM RNA HS Assay Kit (Termo Fisher Scientific, USA). Synthesis of cDNA on a total RNA template (reverse transcription reaction) was performed using the iScript RT Supermix kit (BioRad, USA) on a GNOM thermostat.

The amplification reaction with primers of the genes of interest was carried out using the Maxima SYBR Green/ROX qPCR Master Mix (2x) kit (Termo Fisher Scientific, USA) according to the manufacturer's protocol, the reaction was carried out in standard 96-well optical plates Corning Axygen® PCR-96-LP- FLT-C on Light Cycler® 96 System (Roche, Switzerland). The gene of the TATA-binding protein TBP (housekeeping gene) was used as a reference; since the primer annealing temperature for the studied genes is different, two different housekeeping genes were taken that were suitable for this parameter (TBP and B.actin). Compared to control, the relative fold change in mRNA expression for each gene under study, namely, Ovocalyxin-36 (OCX-36), Ovalbumin (OVAL), Calbindin-1(CALB-1), Ovocleidin 116 (OC-116), Ovocalyxin-32 (OCX-32), avian β-defensin 9 (AvBD-9), gallinacin-10 (Gal-10), interleukin 8 (IL8) and proenkephalin (PENK) were calculated. Table 2 provides the primer sequences and accession numbers for the genes.

**Table 2:** The Primers sequences used in gene expression

Gene	Forward primer (from 5’ to 3’) Reverse primer (from 5’ to 3’)	PCR product size (bp)	Reference
«Housekeeping» genes:			
B.actin	F: ATTGTCCACCGCAAATGCTTC R: AAATAAAGCCATGCCAATCTCGTC	86	[33]
TBP	F: AGCTCTGGGATAGTGCCACAG R:ATAATAACAGCAGCAAAACGCTTG	134	[34]
OVAL	F: AAGACAGCACCAGGACACAGA R: TTCTGGCAGATTGGGTATC	212	[35]
OCX-36	F: TTGGAATGGTCGTCTTCTGTGG R: CGGTCTGAATGATGGCATCG	121	[36]
CALB-1	F: CTCCGACGGCAATGGGTAC R: GGTGTTAAGTCCAAGCCTGCC	96	[37]
OC-116	F: AAGAGCCAACATCCAAGTGGGTGAGAAT R: CAGTGACCACATGGCTCCCTTTCCT	424	[38]
OCX-32	F: GGACAGCACTGCACTACATCAA R: GGAATTTCGTGGAGCAAGACAA	514	[39]
AvBD-9	F:AACACCGTCAGGCATCTTCACA R: CGTCTTCTTGGCTGTAAGCTGGA	131	[33]
Gal-10	F:GCTCTTCGCTGTTCTCCTCT R: CCCAGAGATGGTGAAGGTG	67	[33]
IL8	F:GGAAGAGAGGTGTGCTTGGA R: TAACATGAGGCACCGATGTG	102	[33]
PENK	F:GCTGGATGAGAACCATCTGC R: AGCCTCCGTACCTCTTAGCC		[40]

### Histopathological study

A morphological examination was done for the laying hen’s intestine (jejunum). Tissue samples were collected from five birds/ group and were fixed in 10% neutral buffered formalin for 2–5 days. Samples were dehydrated in ascending grades of ethyl alcohol starting from 50% to absolute. The clearance of the samples was applied using xylene (three changes) and then paraffin impregnation was done in the hot oven using melted paraffin wax (three changes) at 56 °C. Finally, blocks of the processed samples were prepared using paraffin wax and cut using a rotatory microtome. Thin paraffin Sects. (5–7 µm- thick) were cut from the samples’ blocks and mounted on egg albumin-glycerin coated glass slides and dried in an electrical incubator for 30–60 min at 45 °C then stained with Hematoxylin and eosin (H and E) for general inspection of the organ based on Bancroft and Layton [41]. Micrographs of the sections were taken with a digital camera (Leica EC3, Leica, Germany) connected to a microscope (Leica DM500). Jejunum length, depth, crypt, and muscle layer were measured by Image J software (NIH).

### Statistical analysis

Data were analyzed using SPSS version 20.0 (IBM Corp., NY, USA). One-way analysis of variance (ANOVA) was used with subsequent Duncan’s post hoc test.. The overall significance level was set as *p* < 0.05, *p* < 0.01, and *p* < 0.001. All values are expressed as the mean ± standard error. The statistical model is:

$$\mathrm{Xij}=\upmu +\mathrm{Ti}+\mathrm{eij}$$

where Xij = Value of ith observation (the variable such as body weight) of the ith treatment, μ = overall mean, Ti = Effect of ith treatment (Effect of lactulose), and eij = Random error.

## Results

### Egg weight

Effect of Vetelact supplementation in drinking water on egg weight is shown in Table 3. Significant differences among groups started from 36 weeks of age till 39 weeks with heavier egg weight recorded for the three groups supplemented with Vetelact as compared to control group.

**Table 3:** Effect of four successive weeks supplementation of Lactulose (Vetelact) on egg weight (g, mean ± SE) in layer chickens

Egg weight (g)	Treatment groups				*P* value
	**Group 1 (control)**	**Group 2 (vetelact 0.05 ml/kg)**	**Group 3 (vetelact 0.1 ml/kg)**	**Group 4 (vetelact 0.15 ml/kg)**	
**Week 1**	57.90 ± 0.78 ^b^	60.77 ± 0.88 ^a^	60.11 ± 0.74 ^b^	60.63 ± 0.75 ^a^	0.043
**Week 2**	59.18 ± 0.59 ^b^	61.84 ± 0.77 ^a^	61.21 ± 0.63 ^ab^	61.81 ± 0.79 ^a^	0.027
**Week 3**	59.77 ± 0.58 ^b^	62.17 ± 0.75 ^a^	62.57 ± 0.88 ^a^	61.48 ± 0.87 ^ab^	0.024
**Week 4**	59.43 ± 0.76 ^b^	62.02 ± .076 ^a^	62.68 ± 0.92 ^a^	61.88 ± 0.73 ^a^	0.027

Means within a row superscripted by different letters are significantly different (*p* < 0.05)

### Egg production

**Fig. 1:**
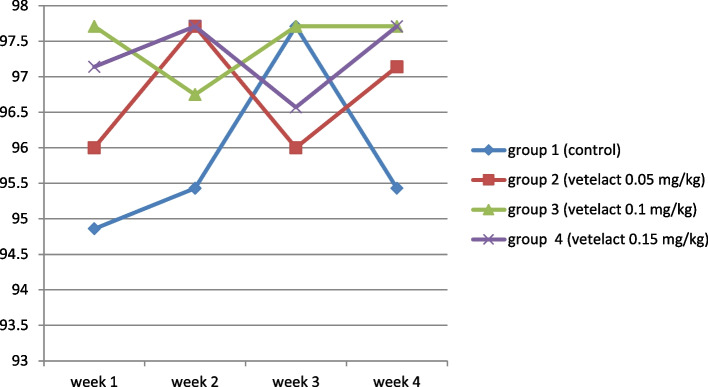
Effect of Lactulose (Vetelact) supplementation for four weeks (weeks 35 to 39) on egg production

Egg production for 4 successive weeks was recorded and presented in Fig. 1. Egg production was not significantly different among groups within the total period of experiment.

### Egg quality

Effect of Vetelact supplementation on egg quality is presented in Table 4. Supplementation of Vetelact did not affect egg index, shell deformation, albumin height, yolk height, and yolk index and Haugh unit among groups. However, yolk weight, shell weight, shell thickness were significantly different with highest values recorded for three groups supplemented with Vetelact when compared to control group, with group 3 (0.10 mg/kg) was the highest value. Yolk diameter was differed among groups with highest diameter recorded in group 3 followed by group 2 and 4, while the least diameter was recorded for control group. Albumin weight was heaviest in Group 2 (38.22 g) and the lightest weight (36.53 g) recorded in control group. Yolk diameter was significantly highest value recorded for group 3 and least value was noticed in control group.

**Table 4:** Effect of four successive weeks supplementation of Lactulose (Vetelact) on internal and external egg quality (mean ± SE) in layer chickens

**Variable**	**Treatment groups**				*P* value
	**Group 1 (control)**	**Group 2 (vetelact 0.05 ml/kg)**	**Group 3 (vetelact 0.1 ml/kg)**	**Group 4 (vetelact 0.15 ml/kg)**	
Egg weight (g)	59.10 ± 0.54^b^	61.54 ± 0.49^a^	61.53 ± 0.63^a^	61.10 ± 0.44 ^a^	0.003
Egg index %	80.27 ± 0.41	80.22 ± 0.39	80.00 ± 0.34	80.45 ± 0.43	0.883
Shell Deformation µm	19.1 ± 0.39	19.48 ± 0.33	19.15 ± 0.39	19.90 ± 0.34	0.385
Yolk diameter (cm)	4.09 ± 0.26^c^	4.17 ± 0.02^ab^	4.21 ± 0.02^a^	4.13 ± 0.02^bc^	0.002
Albumin height(mm)	8.21 ± 0.20	8.41 ± 0.24	8.19 ± 0.27	8.05 ± 0.2	0.748
Yolk height (mm)	20.12 ± 0.20	20.43 ± 0.18	20.56 ± 0.19	20.15 ± 0.16	0.248
Yolk weight (g)	15.44 ± 0.16^b^	15.95 ± 0.2^ab^	16.24 ± 0.19^a^	16.04 ± 0.19^a^	0.018
Shell weight (g)	7.13 ± 0.08^b^	7.38 ± 0.08^a^	7.43 ± 0.09^a^	7.37 ± 0.06^a^	0.034
Albumin weight (g)	36.53 ± 0.44^b^	38.22 ± 0.47^a^	37.86 ± 0.58^ab^	37.68 ± 0.44^ab^	0.041
shell thickness (10^−2^ mm)	38.91 ± 0.41^b^	40.05 ± 0.38^a^	40.91 ± 0.4^a^	40.33 ± 0.34^a^	0.003
Yolk index	0.49 ± 0.004	0.49 ± 0.005	0.48 ± 0.005	0.48 ± 0.003	0.881
Haugh unit	90.51 ± 0.99	90.66 ± 1.26	89.33 ± 1.36	89.03 ± 1.07	0.695

Means within a row superscripted by different letters are significantly different (*p* < 0.05)

### Blood parameters

Effect of vetelact supplementation in feed on blood biochemical components is presented in Table 5. Blood metabolites including glucose, creatinine and urea levels in blood were not statistically different among the groups. However, Vetelact caused significant difference in cholesterol (*P* = 0.040) and total bilirubin (*P* = 0.014) levels among groups. In Group 4 (0.15 ml /kg), it decreased the level of cholesterol (2.22 mmol/L). The least value for total bilirubin (11.33 mmol/L) was in Ggroup 3, while, the highest value of total bilirubin (15 mmol/L) was recorded for Group 1 (Control). Vetelact significantly (*P* = 0.001) decreased the total protein and globulin in serum whereas the lowest level was recorded in Group 4 and the highest level was observed in the control group. However, albumen level did not show any differences among groups. Additionally, albumen/globulin ratio was significantly (*P* = 0.007) different among groups, whereas, the highest ratio (0.53) was observed in Group 2 and 4 while; the least ratio (0.47) was noticed in control group. Phosphorus level was increased significantly (*P* = 0.001) as a result of Vetelact supplementation in Groups 3 and 4 when compared to other groups (Group 2 and Control). However, calcium level was similar in all groups.

**Table 5:** Effect of four successive weeks supplementation of Lactulose (Vetelact) on Blood Biochemical Components (mean ± SE) in layer chickens

**Variable**	**Treatment groups**				*P* value
	**Group 1 (control)**	**Group 2 (vetelact 0.05 ml/kg)**	**Group 3 (vetelact 0.1 ml/kg)**	**Group 4 (vetelact 0.15 ml/kg)**	
**Metabolites. mmol/L**					
Glucose	12.22 ± 0.48	12.42 ± 0.27	12.07 ± 0.17	12.35 ± 0.22	0.386
Creatinine	< 9.00 ± 0.00	< 9.00 ± 0.00	< 9.00 ± 0.00	< 9.00 ± 0.00	
Urea	< 0.60 ± 0.00	< 0.60 ± 0.00	< 0.60 ± 0.00	< 0.60 ± 0.00	
Cholesterol	2.59 ± 0.10 ^ab^	2.67 ± 0.27 ^ab^	2.87 ± 0.20 ^a^	2.22 ± 0.06 ^b^	0.040
TBIL	15.00 ± 0.50 ^a^	11.33 ± 0.73 ^b^	11.33 ± 1.36 ^b^	12.00 ± 0.58 ^b^	0.014
**Proteins. g/L**					
TP	62.00 ± 1.32^a^	57.67 ± 1.42^b^	56.67 ± 0.60^bc^	54.33 ± 0.60^c^	0.001
Albumin	20.33 ± 0.33	19.67 ± 0.17	19.67 ± 0.44	19.33 ± 0.44	0.281
Globulin	42.00 ± 1.32^a^	38.00 ± 1.26^b^	37.00 ± 0.29^b^	35.33 ± 0.17^b^	0.001
ALB/GLOB	0.47 ± 0.02^b^	0.53 ± 0.02^a^	0.50 ± 0.00^ab^	0.53 ± 0.02^a^	0.007
**Minerals. mmol/L**					
Phosphorus	2.08 ± 0.14^b^	2.08 ± 0.15^b^	2.98 ± 0.08^a^	2.61 ± 0.25^a^	0.001
Calcium	> 4.00 ± 0.00	> 4.00 ± 0.00	> 4.00 ± 0.00	> 4.00 ± 0.00	
**Enzymes. U/L**					
ALT	< 10.00 ± 0.00	< 10.00 ± 0.00	< 10.00 ± 0.00	< 10.00 ± 0.00	
ALKP	114.33 ± 14.86^b^	186.00 ± 30.09^a^	224.67 ± 5.53^a^	199.67 ± 20.42^a^	0.003
GGT	35.00 ± 2.75^ab^	33.00 ± 1.61^ab^	30.33 ± 2.17^b^	37.67 ± 2.19^a^	0.140
Amylase	211.67 ± 12.72	233.67 ± 14.82	237.67 ± 8.84	213.67 ± 7.50	0.258
Lipase	69.67 ± 4.44^c^	107.00 ± 2.65^a^	86.33 ± 1.67^b^	90.67 ± 9.59^b^	0.001

Means within a row superscripted by different letters are significantly different (*p* < 0.05)

*TP* total protein, *TBIL* Total bilirubin, *ALB* Albumin, *GLOB* Globulin, *ALT* alanine transaminase, *ALKP* Alkaline phosphatase, *GGT* gamma-glutamyl transferase

Effect of Velelact on enzymes is presented in Table 5. Alanine transaminase and amylase enzymes did not differ among the groups. However, Vetelact significantly increased the level of alkaline phosphatase with highest level recorded for 0.10 mg /kg (224.67 U/L) and the least level recorded for control group (114.33 U/L). Moreover, gamma-glutamyl transferase differed significantly among groups with group 4 (0.15 mg /kg) as highest level followed by the control group then the least level in group 3 (0.1 mg /kg). Group 2 showed the highest level of lipase enzyme (107 U/L) followed by Group 3, 2 then the lowest value was for control group (69.67 U/L).

### Gene expression

**Fig. 2:**
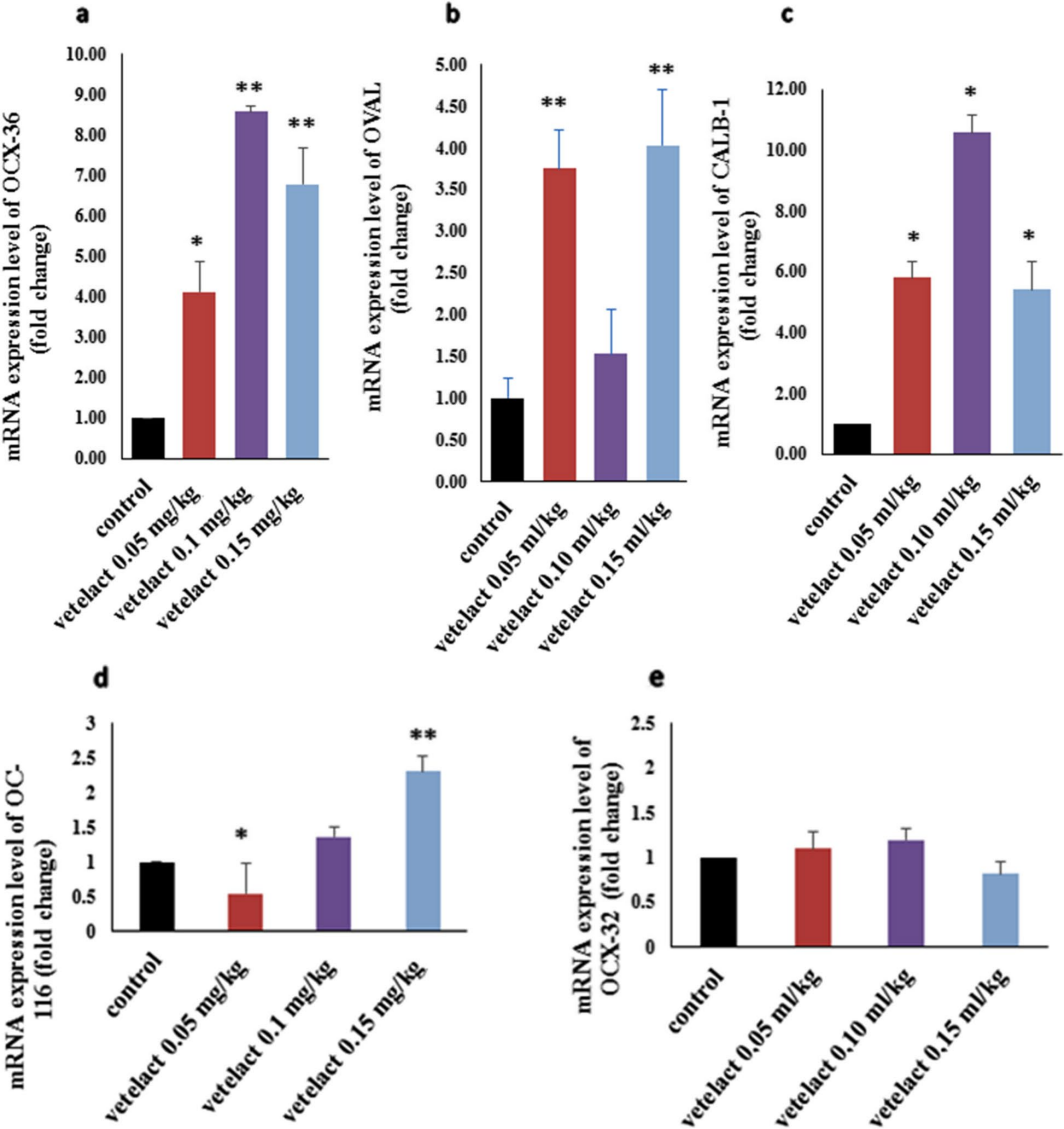
Effect of Lactulose (Vetelact) treatments (0.05, 0.10 and 0.15 ml/kg of body weight) in drinking water on mRNA expression of OCX-36 (**a**), OVAL (**b**), CALB-1 (**c**), OC-116 (**d**) and OCX-32. The data represented as means ± SE. * and ** indicates *p* < 0.05 and *P* < 0.01

**Fig. 3:**
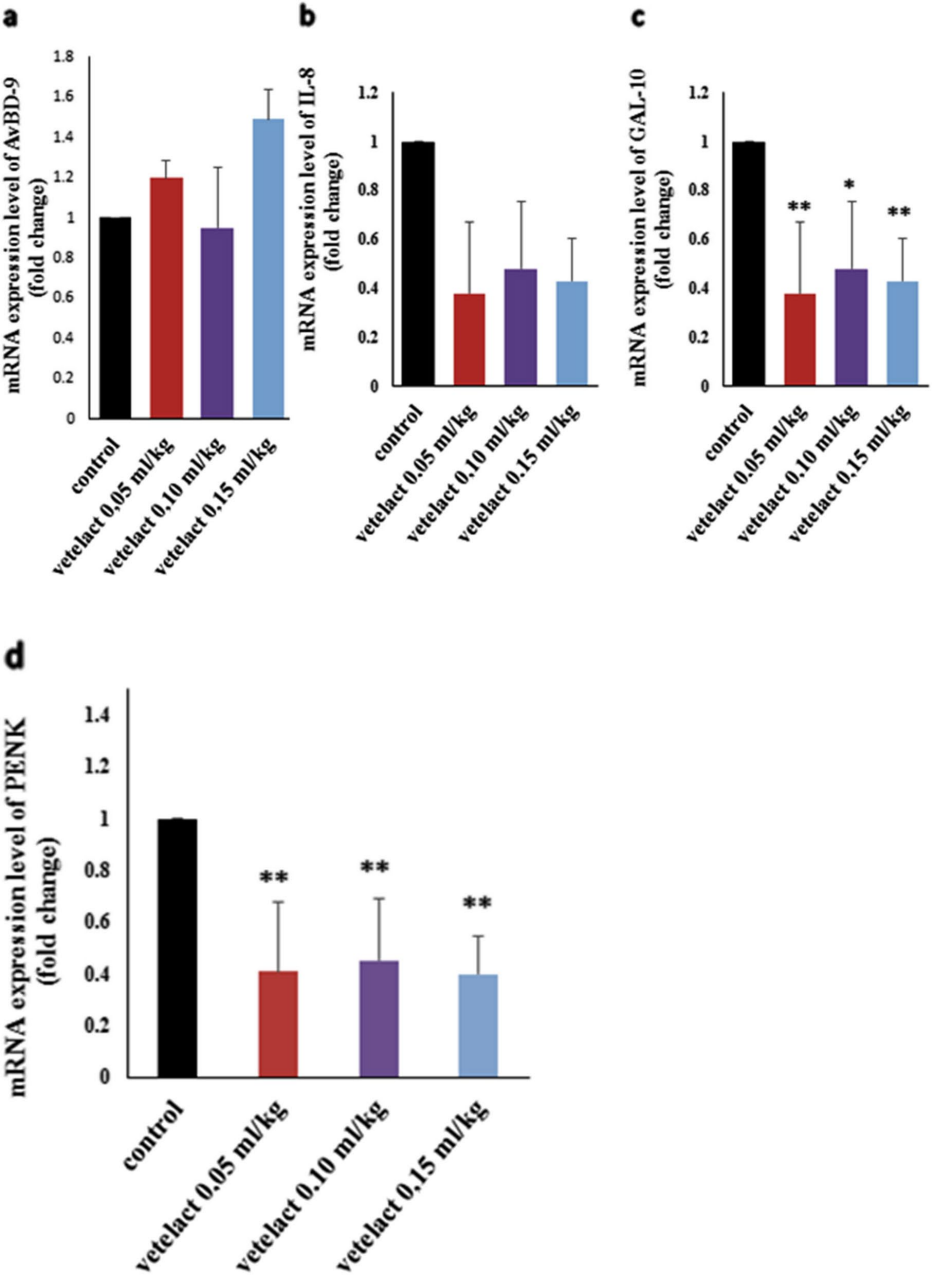
Effect of Lactulose (Vetelact) treatments (0.05, 0.10 and 0.15 ml/kg of body weight) in drinking of laying hens water on mRNA expression of AvBD-9 (**a**), IL-8 (**b**), GAL-10 (**c**), PENK (**d**) and NRF-2. The data represented as means ± SE. *, ** and *** indicates *p* < 0.05, *P* < 0.01 and *P* < 0.001

Figures 2 and 3 show that in comparison to the control group, supplementation with three Vetelact (0.05, 0.10 and 0.15 ml/kg body weight) treatments upregulated the OCX-36, OVAL and CALB-1 but downregulated the mRNA expression of Gal-10 and PENK; additionally, Vetelact at a dose of 0.05 ml/ kg body weight downregulated the OC-116 (Fig. 2d) while the other two doses (0.10 and 0.15 ml/ kg body weight) upregulated the gene expression. The mRNA expression of OCX-32 (Fig. 2e) and IL8 (Fig. 3b) genes were upregulated in response to Vetelact at doses of 0.05 and 0.10 ml / kg body weight, while downregulated in response to Vetelact at dose of 0.15 ml/kg body weight when compared to control group. Furthermore, the mRNA expression of AvBD-9 (Fig. 2e) was upregulated in response to the dose of 0.05 and 0.15 while it was downregulated at 0.10 ml/ kg body weight of Vetelact as compared to the control group.

### Histopathologic findings

**Fig. 4:**
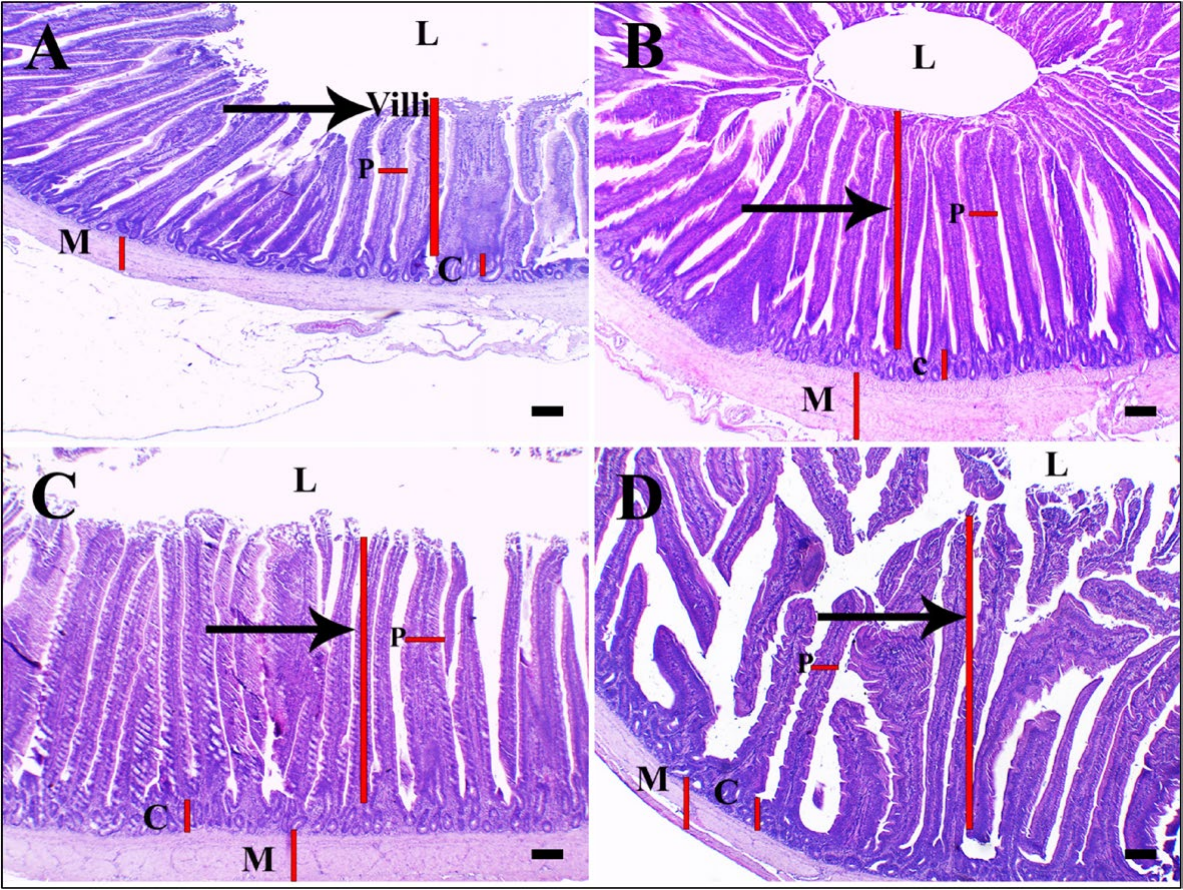
Effect of Lactulose (Vetelact) treatments (0.05, 0.10 and 0.15 ml/kg of body weight) in drinking water of laying hens on Photomicrograph small intestine (jejunum). Tissue stained by hematoxylin and eosin. Scale bar = 200 µm. **A** control group showing normal intestinal villi length (arrow, red line), propria width (P), crypts (C), lumen (L) and muscle layer (M). **B** the group supplied by 0.05 ml/kg showing increased in villi length (arrow, red line), crypts (C), P, and thick muscle layer (M). **C** the group supplied by 0.1ml/kg showing villi with longer length (arrow, red line), deep crypts, p, and muscle layer. **D** the group supplied by 0.15 ml/kg showing higher villi length (arrow), wide propria, crypts (C), and muscle layer (M)

**Fig. 5:**
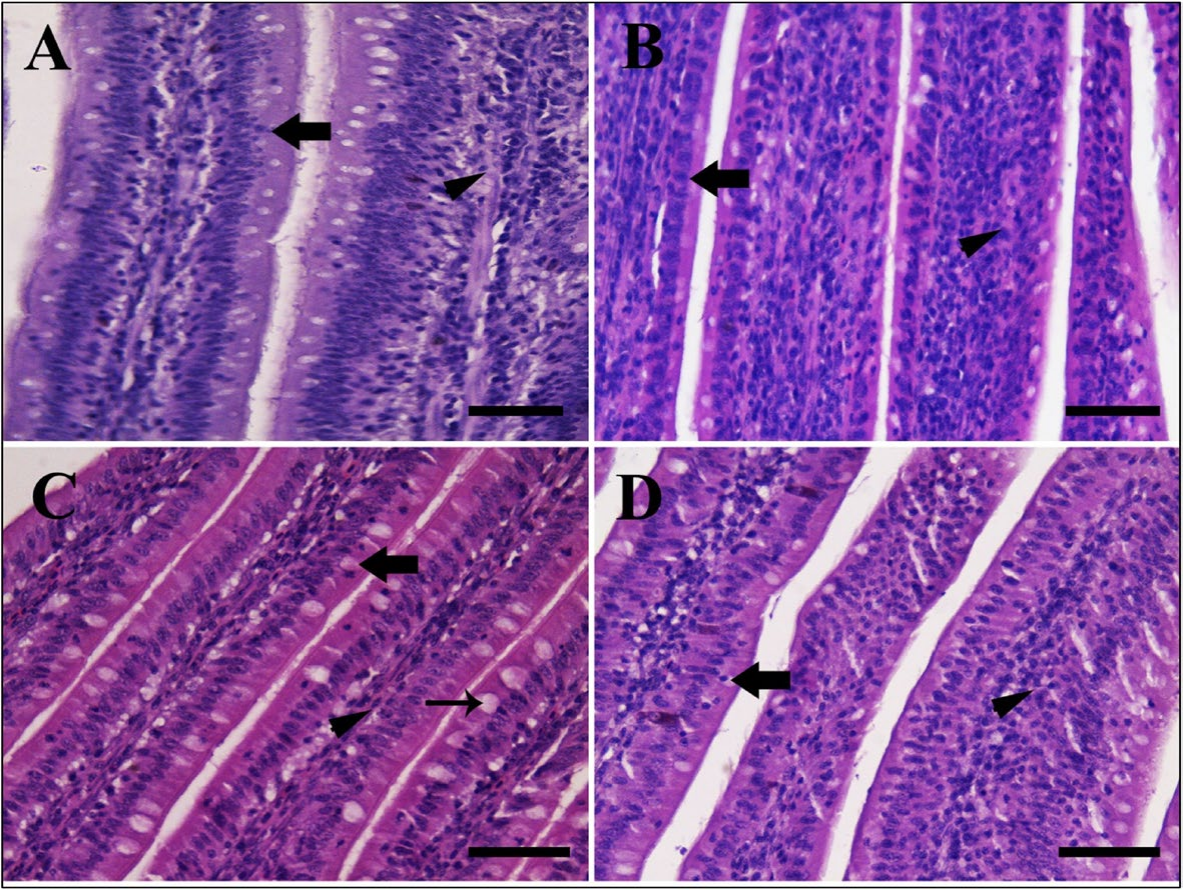
Effect of Lactulose (Vetelact) treatments (0.05, 0.10 and 0.15 ml/kg of body weight) in drinking water of laying hens on Photomicrograph small intestine (jejunum). Tissue stained by hematoxylin and eosin. Scale bar = 50 µm. **A** control group showing normal intestinal villi lining epithelium (arrow) and lamina propria (arrowhead). **B** group supplied by 0.05 ml/kg showing slightly improved epithelial lining with mucus covering (arrow) and wide lamina propria with a proliferation of cells (arrowhead). **C** showing group with 0.1ml/kg treatments, improved lining epithelium (thick arrow), more goblet cells (clear space, thin arrow) with higher mucous coating the epithelial surface and lamina propria with proliferation of cells (arrowhead). **D** group treated by 0.15 ml/kg showing lining epithelium with decreased goblet cell distribution (arrow) and wide lamina propria (arrowhead)

**Fig. 6:**
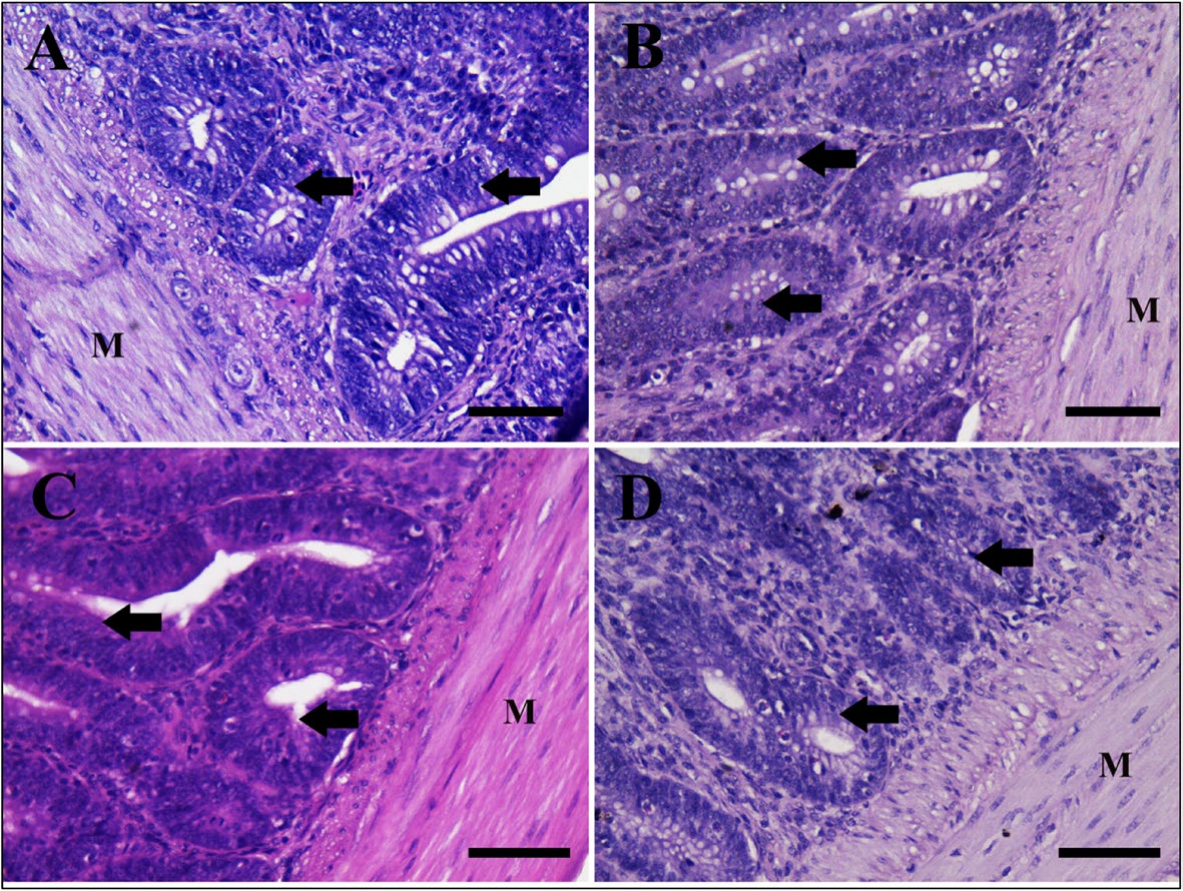
Effect of Lactulose (Vetelact) treatments (0.05, 0.10 and 0.15 ml/kg of body weight) in drinking water of laying hens on Photomicrograph small intestine (jejunum). Tissue stained by hematoxylin and eosin, Scale bar = 50 µm. **A** control group with normal intestinal crypts, glands (arrows), tunica muscularis layer of smooth muscle fiber (M). **B** group treated by 0.05 ml/kg, showing normal crypts (arrows) and thick well- developed muscle layer (M). **C** group treated by 0.1ml/kg showing improved intestinal crypts (arrows) and thick muscle layer (M). **D** a group treated by 0.15 ml/kg showing normal crypts (arrows) and muscle layer (M)

Table 6 and Figs. 4, 5 and 6 show the histopathologic finding. By histopathological examination, the small intestine (Jejunum) of chicken is involved in the absorption of the bulk of nutrients [42]. The normal structure of jejunal villi is longer finger-like projections that extend into the lumen of the small intestine and are lined by simple columnar epithelium with few goblet cells located in between [43]. Below the epithelium, the lamina propria is a loose and very cellular irregular connective tissue. Most of the cells within the meshes of the collagen fibrils are plasma cells, although many other cell types (including almost all of the true cells of the blood) can be found. The intestinal crypts are located between the villi and extend deep into the tunica mucosa [44]. The outer layer is the *tunica muscularis* which is formed from the inner circular and outer longitudinal of smooth muscle (Figs. 4A, 5A, and 6A).

**Table 6:** Histomorphometric analysis of intestine from layer chickens supplemented with four successive weeks of Lactulose (Vetelact)

Measurement (μm)	Treatment groups				*P* value
	**Group 1 (control)**	**Group 2 (vetelact 0.05 ml/kg)**	**Group 3 (vetelact 0.1 ml/kg)**	**Group 4 (vetelact 0.15 ml/kg)**	
**Villi height**	747.16 ± 8.91^c^	957.37 ± 13.99^b^	1084.79 ± 9.09^a^	1098.02 ± 12.78^a^	< .0001
**Villi width**	66.79 ± 2.87^c^	80.34 ± 3.14^b^	70.27 ± 2.28^c^	91.79 ± 1.16^a^	< .0001
**Crypt depth**	107.00 ± 6.11^c^	142.49 ± 7.11^b^	179.52 ± 8.42^a^	150.99 ± 1.52^b^	< .0001
**Tunic muscularis thickness**	157.46 ± 3.20^c^	254.61 ± 18.12^a^	200.74 ± 0.37^b^	126.52 ± 0.88^d^	< .0001

Data are presented as Means ± SE. Those within a row superscripted by different letters are significantly different (*p* < 0.05)

Villi increase the internal surface area of the intestinal walls making available a greater surface area for absorption. An increased absorptive area is useful because digested nutrients (including monosaccharides and amino acids) pass into the semipermeable villi through diffusion, which is effective only at short distances. In other words, increased surface area (in contact with the fluid in the lumen) decreases the average distance traveled by nutrient molecules, so the effectiveness of diffusion increases. The villi are connected to the blood vessels so the circulating blood then carries these nutrients away (Fig. 5A).

Vetelact prebiotic improved the villi available for absorption, increased mucous production by goblet cells in epithelial lining, the proliferation of cells in lamina propria, increased blood supply to the epithelial lining and induced development of intestinal crypts for production bulk of mucous and intestinal hormone which help in absorption as well as improved the *tunica muscularis* layer of smooth muscle which help the movement of nutrient in the intestine for absorption. So, we tested different Vetelact doses evaluate its effects on intestinal absorption surface area; beneficial effects were evidence from low dose of 0.05 ml/kg which showed mild increase in intestinal villi length with improved lining of epithelium, lamina propria with the proliferation of cells, intestinal crypts and increase the thickness of muscle layer ( Figs. 4B, 5B, and 6B).

Using 0.10 ml/kg showed a higher increase in villi length (Table 6) with more mucus covering epithelial cells, an increase in goblet cells number within the lining epithelium, proliferation of cells in lamina propria, improvement of intestinal crypts with population of cells and thick *tunica muscularis* layer (Figs. 4C, 5C, and 6C).

Using 0.15 ml/kg showed little changes in epithelial lining than a control group with increased intestinal villi length similar to the previous group with normal lining cells and goblet cells, increase crypts depths with normal lining cells, wide propria with cells proliferation and adjustment of muscle layer for absorption (Figs. 4D, 5D, and 6D).

## Discussion

Prebiotics such as lactulose are substrates that are selectively utilized by host microorganisms to confer health benefits [45]. Hens laying rate is positively impacted by prebiotics. Previous studies have shown that Lactulose has been proven to have a positive impact on pig breeding and poultry production [28, 29, 46]. Other feeding trials demonstrated that addition of lactulose in the diets of laying hens and broilers leads to significant improvements in egg production rate, average body weight, feed conversion ratio, and enhanced immunity (23, 28, 47). In the present study, supplementation of birds with Vetelact (0.05, 0.10 and 0.15 ml/kg live body weight) improves egg production at the end of experiment (*P* > 0.05) when compared to control. Vetelact also significantly increased egg weight. The increase in egg weight in Vetelact supplemented groups probably due to heavier yolk and shell weights when compared to the control group. The beneficial effect of Lactulose on egg production in this study may also be linked to the overexpression of genes associated to reproduction, including OVAL and OCX-36 in uterus of laying hens. In line with our study, Vetelact (0.10 ml/kg of body weight) had a positive prolonged effect on laying performance of the laying hens aged 56–59 weeks, as well as positive effects on the intestinal microflora with increased numbers of *bifidobacteria* and cellulolytic bacteria in the intestine and reduced the total number of pathogenic and undesirable microflora [47]. These beneficial effects could be attributed to the active components of Vetelact (i.e., lactulose and lactose) which have been shown to have antibacterial and anti-inflammatory properties and to improve gut health and nutrient utilization [12, 13]. Similarly, laying rate has been improved by using prebiotics as recorded by previous studies [48, 49]. Additionally, use of the prebiotic oligofructose and inulin in layer diets increased egg weight by 12.5 and 11%, respectively. Egg production increased by 13.4 and 10.7%, respectively compared with the control [23]. However, Kochish et al. [50] reported that Vetelact did not have a significant effect on egg production and even led to some decrease in comparison with the control in laying hens. These contrasts may be attributed to prebiotic supplementation doses, the type of laying hen, the feeding phase, and environmental factors.

Egg quality is an important parameter as it influences the hatchability and economic profitability of egg production [51]. Eggshell thickness is a crucial factor in egg transportation and storage and is a key predictor of egg quality [52]. The availability of intestinal calcium is essential for eggshell calcification because it is essential for supplying enough calcium to meet shell quality requirements [53].

Stronger eggshells are a result of higher calcium absorption [23]. Dietary prebiotics improve eggshell quality [54]. In the current study, there were significant effects of Vetelact supplementation on shell thickness, shell weight, yolk weight and albumen weight as compared to control. Similarly, Inulin and synbiotic increased eggshell thickness and eggshell calcium content and lowered eggshell deformations [55]. The positive effect of Lactulose on eggshell quality in the present study can be attributed to the upregulation of the CALB-1 gene expression. The CALB-1 gene plays a crucial role in the biomineralization process of the eggshell, leading to improvements in its overall egg quality.

In the present study, Vetelact treatments did not affect blood biochemical components as glucose, creatinine, urea, albumin, calcium, ALT and amylase by. However, Vetelact (0.05, 0.10 and 0.15 ml/ kg body weight) decreased total bilirubin, total protein and globulin as compared to control. Additionally, all treatments of Vetelact increased the level of ALKP and lipase as compared to control. Vetelact (0.10 and 0.15 ml/ kg body weight) also increased the level of phosphorus. Similarly, adding lactulose in ration caused increase in phosphorus level in rabbit [56]. Adding of lactulose to broiler diet led to an increase in the serum level of urea, glucose, phosphorus, ALT, total protein and albumin [26].

Our results showed decrease in cholesterol level in blood as a response to Vetelact (0.15 ml/kg body weight) when compared to other groups. Similarly the use of prebiotic in broiler diet, decreased (*P* < 0.05) the serum cholesterol level on day 35 as compared with the control [57]. The most important way of cholesterol excretion is through bile acids produced in the liver [58]. It is possible to lower cholesterol levels by using probiotics and prebiotics to break down bile salts, de-conjugate the production of enzymes by lactic acid bacteria, and lower the pH in the intestinal tract. Low pH reduces the solubility of non-conjugate bile acids, which causes them to be absorbed less from the intestine and expelled more in the faeces [59]. As a result, the liver converts greater cholesterol concentration into tissues in order to re-establish the hepatic cycle of bile acids, which lowers the levels of cholesterol in the blood [60].

In this study, gene expression in the shell gland of laying hens was investigated after supplementation with different doses of lactulose-based Vetelact. The reproductive hormones play a significant role in stimulating egg formation and yolk ovulation, particularly during the active calcification stage. These hormones are crucial in regulating calcium metabolism [61]. Additionally, certain genes involved in the biomineralization process and/or the supply of shell precursors might undergo upregulation due to the lactulose supplementation. The primary focus of this research was on genes potentially involved in the calcification process. Previous studies have shown interactions between these genes and crystal formation [62]. However, the effects of lactulose on the expression of reproductive genes were not thoroughly investigated. The physical quality aspects of eggs, such as shell thickness, egg shape, and elasticity, are influenced by the mRNA expression of OC-116 in conjunction with OCX-32 genes [63, 64]. The absence of these matrix proteins can lead to the complete cessation of the calcification process [65]. Irregular expression of the OC-116 gene has been associated with fragile, misshapen, and thin eggshells [66]. Ovocleidin-116 is a significant element of the chicken eggshell matrix that observed in the palisade layer and it is most abundant in uterine fluid during the intense eggshell calcification phase. It is only expressed in the uterus and is believed to be primarily responsible for controlling the eggshell calcification [67]. Ovocalyxin-32 (32kDa), which is mostly prevalent during the terminal phase of calcification and is consequently located in the outer portion of the eggshell, is present in uterine fluid during the growth phase [39]. Ovocalyxin-36 is expressed only in uterine tissue and its expression is significantly upregulated after the egg enters the uterus. This protein is therefore a viable candidate for regulating shell formation [36].

We evaluated the effect of Vetelact supplementation in drinking water with (0.05, 0.10 and 0.15 ml/kg) on gene expression associated with reproduction and immunity in layers. Vetelact supplementation increased the mRNA expression of the OCX-36, OVAL, CALB-1 and OC-116 of layers in a dose-dependent manner (0.10 and 0.15 ml/kg), indicating its positive effect on reproductive performance. Similarly, Muhammad et al. [38] found that regardless of dietary Se treatments, mRNA expression of OCX-32 and OCX-36 was up-regulated in the shell gland. Similarly, addition, Jonchère et al. [68] established that OCX-36 is shell gland specific, and increases through the calcification of eggshell.

The ovalbumin gene (OVAL) encodes ovalbumin of egg white [69]. The synthesis of ovalbumin affects both the egg mass directly and the time of passage of the follicle through the oviduct, that is, indirectly on the number of eggs laid [70].

In avian species, Calbindin is found in the intestine and eggshell gland tissues that are characterized by massive transfer of Ca^+2^ [71] where, calbindin and Ca^2+^ transport is closely correlated [72]. Calbindin is a 28 kDa calcium-binding protein, which fluctuates in a circadian fashion during the daily egg cycle, in close temporal association with eggshell calcification [73]. Calbindin expression is related to eggshell quality [74]. In the eggshell gland, calbindin appears during the formation of the first eggshell at the onset of egg production and disappears within three days of its cessation [75]. The concentration of calbindin in the eggshell gland is proportional to the rate of shell Ca^2+^ deposition [76]. In addition to their supposed function in Ca^2+^ transport, calbindins may also play a protective role in cells' resistance to high Ca^2+^ concentrations or the cellular degeneration caused by apoptosis and may also act as a buffer [77]. In a study conducted by Sun et al. [78], it was found that the expression of the CALB-1 gene in the uterus of the strong shell group was significantly higher compared to the weak shell group. These results are in agreement with our fining that the lactulose supplementation significantly upregulated CALB-1 gene in uterus of laying hens this may explain the improvement in eggshell thickness.

The biologically active additives investigated had a complex impact on the functional activity of immune-related genes (AvBD9, IL8, PENK, GAL-10). Supplementation of prebiotic Vetelact led to a significant decline (5.0 times) in the expression of AvBD9 and IL8 genes [50]. These results are in agreement with our results, Vetelact supplementation (0.15 ml/kg body weight) markedly reduced IL-8 mRNA expression. Our results revealed that there was significant downregulation of GAL-10 and PENK genes mRNA in the layers supplemented with Vetelact (*p* < 0.05) compared to their control. Similarly, in *ovo* injection of prebiotic inulin resulted in downregulation of Interleukin (IL)-4, IL-6, IL-8, IL-12p40, and IL-18 in the cecal tonsils and spleen [79]. On the other hand, there were no significant differences for gene expressions of immune responses IL-6, IL-10 and IFN-γ on d 21 and 35 after application of MOS and β-glucan in broiler chickens [80]. Prebiotics in chicken feeds boost avian immunity by supporting the production of beneficial microbes in a targeted manner. This mechanism increases the manufacture of a number of chemicals, including bacteriocins and SCFA, which, in addition to inhibiting pathogen development, play a role in the signaling pathway of the immune system [81].

By histopathological examination, Villi increase the internal surface area of the intestinal walls making available a greater surface area for absorption. In our results, Vetelact supplementation increased the villi length that will subsequently improve the absorption, increases mucous production by goblet cells in lining epithelium, the proliferation of cells in lamina propria, increases blood supply to the epithelial lining and induce development of intestinal crypts for production bulk of mucous and intestinal hormone that help in absorption as well as improved the *tunica muscularis* layer of smooth muscle that help the movement of nutrient in the intestine for absorption.

The villi length, lamina propria layer, intestinal crypts depth as well as the *tunica muscularis* layer were improved as a dose (0.05 and 0.10 ml / kg body weight) dependent manner in response to Vetelact treatments. Similarly, Villus width and villus surface area in jejunum had increased linearly with the increasing level of lactulose in broilers on d 42 [20]. Additionally, *in ovo* injection of the prebiotic DiNovo increased surface area of the intestinal villi as compared to control in duodenum of broiler chickens at 21 and 42 day [82]. An increase in height of intestinal villi and the appropriate ratio between the height of villi and crypt depth are a measure of the intensity of recovery processes of intestinal epithelial cells. This result is consistent with Samanya and Yamauchi, [83]. However, in the present study, using a higher dose of 0.15 ml/kg of Vetelact showed a slight increase in the villi length and a decrease crypts depth as well as smooth muscle layer than the group supplemented with 0.10 ml/kg. The shortening of the villi and deepening of crypts may reduce the productivity of the flock because shorter villi reduce the total surface area of the intestinal absorption which results in poorer absorption of nutrients, and deeper crypt contributing to increased secretion of digestive enzymes [84]. So we recommend adding Vetelact at dose of 0.10 ml/ kg body weight.

## Conclusion

This study demonstrates the effects of using different doses (0.05, 0.10, and 0.15 ml/kg) of Vetelact prebiotic in drinking water for four weeks in layer hens. Vetelact supplementation improved egg weight, egg production as well as egg quality. It also decreased the total bilirubin, total protein and globulin, increased the level of ALKP, lipase and phosphorus as compared to control. Vetelact (0.10 ml/kg body weight) upregulated the OCX-36, OVAL, CALB-1, OC-116, OCX-32 and IL8 downregulated the mRNA expression of Gal-10, PENK and AvBD-9. The best histomorphology for intestine, with increased in the villi length and depth of the intestinal crypts with more goblet cell distribution and obvious mucus covering on the surface, was observed at a dose of 0.10 ml/kg.

## Data Availability

The datasets during the current study available from the corresponding author on reasonable request.
